# The co-development and evaluation of an e-learning course on spinal cord injury physical activity counselling: a randomized controlled trial

**DOI:** 10.1186/s12909-024-05141-7

**Published:** 2024-03-06

**Authors:** Femke Hoekstra, Heather L. Gainforth, Rogier Broeksteeg, Stephanie Corras, Delaney Collins, Electra Eleftheriadou, Sonja Gaudet, Emily E. Giroux, Laura S. Kuipers, Shannon McCallum, Jasmin K. Ma, Erica de Passillé, Diane Rakiecki, Shannon Rockall, Rita van den Berg-Emons, Anniek van Vilsteren, Megan Williamson, Jereme Wilroy, Kathleen A. Martin Ginis

**Affiliations:** 1 School of Health and Exercise Sciences, The University of British Columbia, Kelowna, BC Canada; 2 International Collaboration on Repair Discoveries (ICORD), The University of British Columbia, Vancouver, BC Canada; 3 Rijndam Rehabilitation Institute, Rotterdam, The Netherlands; 4 School of Kinesiology and Health Studies, Queen’s University, Kingston, ON Canada; 5 School of Occupational Therapy, Dalhousie University, Halifax, NS Canada; 6 Centre for Teaching and Learning, The University of British Columbia, Kelowna, BC Canada; 7 Spinal Cord Injury British Columbia, Vancouver, BC Canada; 8 The Thompson Okanagan Tourism Association, Vernon, BC Canada; 9 VU University Amsterdam, Amsterdam, the Netherlands; 10 Therapeutic Recreation Program, St. Lawrence College, Kingston, ON Canada; 11 Arthritis Research Canada, Vancouver, BC Canada; 12 School of Kinesiology, University of British Columbia, Vancouver, BC Canada; 13 Horizon Health Network, Stan Cassidy Centre for Rehabilitation, Fredericton, NB Canada; 14 Praxis Spinal Cord Institute, Vancouver, BC Canada; 15 Access Community Therapists, Vancouver, BC Canada; 16 Department of Rehabilitation Medicine, Erasmus MC, University Medical Center Rotterdam, Rotterdam, the Netherlands; 17 Vogellanden Revalidatie Centrum, Zwolle, the Netherlands; 18 Ocean Rehab and Fitness, Vancouver, BC Canada; 19 Department of Physical Medicine and Rehabilitation, University of Alabama at Birmingham, Birmingham, AL USA; 20 Department of Medicine, Division of Physical Medicine & Rehabilitation, The University of British Columbia, Vancouver, BC Canada; 21 Centre for Chronic Disease Prevention and Management, The University of British Columbia, Kelowna, BC Canada

**Keywords:** E-learning course, Physical activity counselling, Health professionals, Spinal cord injury, Randomized controlled trial, Mixed-methods

## Abstract

**Background:**

Health, fitness and lifestyle professionals can play important roles in promoting physical activity in groups at risk of developing an inactive lifestyle, such as people with spinal cord injury (SCI). Tailored counselling is a promising tool to promote and improve physical activity levels. To support professionals to effectively have a conversation about physical activity with clients with SCI, evidence-based training and resources are needed. This project aimed to (1) co-develop an e-learning course on best practices for SCI physical activity counselling and, (2) examine the effectiveness and usability of this course.

**Methods:**

Guided by the technology-enhanced learning (TEL) evaluation framework, we used a systematic, multistep approach to co-develop and evaluate an e-learning course. The development process was informed by input and feedback from a diverse group of end-users and experts (*n* > 160) via online surveys and (think-aloud) interviews. A randomized controlled trial was used to compare learning outcomes (post-knowledge and self-efficacy) between participants who completed the course (intervention group) and the wait-listed control group. Usability, learning experiences, and satisfaction were assessed among all participants.

**Results:**

Forty-one participants (21 intervention-group; 20 control-group) with various backgrounds (e.g., lifestyle counsellors, physiotherapists, occupational therapists, recreation therapists, fitness trainers) enrolled in the randomized controlled trial. After completing the course, participants in the intervention group showed significantly improved knowledge on the best practices for SCI physical activity counselling and higher self-efficacy for using these best practices in conversations with clients with SCI compared to the control group (*p* <.001). Participants reported above average usability scores, positive learning experiences, and high levels of satisfaction when completing the course.

**Conclusion:**

We used a systematic, multi-step, theory-informed approach to co-develop and evaluate an evidence-based e-learning course on SCI physical activity counselling to support professionals to promote physical activity in their daily practices. The overall positive findings demonstrate that the e-learning course is feasible and ready for further implementation in various health and community settings. Implementation of the e-learning course can help professionals improve the physical activity support they provide to their clients, and subsequently increase physical activity participation in people with SCI.

**Supplementary Information:**

The online version contains supplementary material available at 10.1186/s12909-024-05141-7.

## Background

Health, fitness and lifestyle professionals can play important roles in promoting and increasing physical activity participation among their clients [1, 2]. Physical activity promotion is particularly important among groups who are at risk for developing an inactive lifestyle, such as people with spinal cord injury (SCI). To illustrate, physical activity participation in people with SCI is low compared to people without disabilities [3, 4] and compared to people with other types of disabilities [5]. These low physical activity levels can be explained by the many and unique barriers reported by this population [6, 7]. Professionals, such as lifestyle counsellors, physiotherapists, occupational therapists, recreation therapists, SCI peer mentors, and fitness trainers, can support their clients with SCI to become and stay physically active via counselling.

Physical activity counselling can be defined as any type of behavioral support (conversation) on starting, changing, and/or maintaining a physically active lifestyle [8]. These conversations can be part of a therapy session, a fitness program, or peer mentorship program. To support professionals in having these physical activity conversations with their clients with SCI, an international panel of researchers, counsellors and people with SCI co-created theory- and evidence-based best practices for SCI physical activity counselling [8]. These best practices provide guidance on how to have a conversation about physical activity and what to say during a conversation. To effectively use these best practices, professionals need sufficient knowledge, skills and confidence on how to use the best practices in their daily routines. As such, additional training resources are needed to support professionals to use the best practices in physical activity conversations with clients with SCI.

E-learning can be a useful and effective way to improve SCI physical activity counselling knowledge and confidence among a large, diverse group of health, fitness and lifestyle professionals, including those working in rural and remote communities. The benefits of e-learning courses are the flexibility in when and how to access learning material, a standardized way of delivering course content, cost-effectiveness compared to in-person training opportunities, and options for personalized learning [9, 10]. Furthermore, e-learning courses can be created in an interactive, accessible and engaging way by including combinations of text, audio- and video-recordings, quizzes, exercises and links to other resources, using Universal Design for Learning (UDL) Guidelines (e.g., multiple means of representation) [11]. As such, e-learning courses can provide an efficient way for professionals to continue their education and stay up-to-date in their field.

Despite these benefits of e-learning courses for professionals, there are also potential downsides that should be considered. Examples of downsides include limited interaction, technical issues and difficulties, lack of social support, and a requirement of high levels of self-motivation and self-discipline [9, 10]. For example, low perceived usability and technical difficulties can affect users’ learning motivations, experiences and outcomes [10]. Developing an effective, user-friendly, accessible e-learning course that meets the needs and preferences of end-users is complicated and takes time. It requires the engagement of potential end-users early and throughout the development process. Furthermore, systematic evaluations of new e-learning courses are important to examine usability of the course, assess learning outcomes and explore users’ learning experiences prior to large-scale implementation.

Various theoretical frameworks exist to guide the development and evaluation of e-learning resources [12–15]. An example of such a framework developed in the context of medical education is the technology-enhanced learning (TEL) evaluation framework [15]. The TEL-framework is developed by building on existing learning models, including the Context, Input, Process, Product (CIPP) evaluation model [13, 14] and Kirkpatrick model [12]. The TEL-framework outlines seven areas of evaluation activities: conduct needs analysis and environmental scan; document processes, decisions, and final product, test usability, document key events during implementation and final product, assess participant experience and satisfaction, and assess learning outcomes, and estimate cost, reusability, and sustainability. Due to its focus on both the development and evaluation activities of e-learning resources, the TEL-framework provides an ideal framework to guide the co-development and evaluation of an e-learning course for health, fitness and lifestyle professionals on SCI physical activity counselling. Such a course could support professionals and other individuals to promote physical activity in their daily practices and have effective conversations about physical activity with their clients with SCI.

Guided by the TEL-framework, this project aimed to: (1) co-develop an e-learning course on best practices for SCI physical activity counselling and, (2) examine the effectiveness of this course. More specifically, the second aim (i.e., the evaluation study) focused on assessing learning outcomes (i.e., post-knowledge and self-efficacy), testing usability and feasibility, and examining participants’ experiences and satisfaction of completing the e-learning course on SCI physical activity counselling.

## Methods

### Project overview and partnership

This project included two parts. Part 1 focused on the co-development process of the e-learning course, and part 2 focused on the evaluation of the e-learning course. The reporting of this paper is guided by the seven areas of the evaluation activities of the TEL-framework [15] and the Criteria for Reporting on Development and Evaluation of Professional Training interventions in Healthcare (CRe-DEPTH) [16] (refer to Appendix 1). The evaluation study (randomized controlled trial) was registered with the ISRCTN registry on 17/11/2023 (registration number: ISRCTN15500262). The reporting of the trial was guided by the CONSORT reporting guidelines [17].

An international expert panel (i.e., SCI Physical Activity Counselling Panel) was established to co-develop and evaluate the e-learning course. The panel included SCI researchers, counsellors, physiotherapists, occupational therapists, recreation therapists, personal trainers, inclusive education expert, and people with SCI from Canada, the United States of America, and the Netherlands. Appendix 2 provides information on the expertise and background of panel members as well as group-level demographics. The majority of the panel members (83%) were also involved in the co-creation of the best practices for SCI physical activity counselling [8]. Additional details on the selection of panel members and considerations are described elsewhere [8]. We used an Integrated Knowledge Translation (IKT) approach to co-develop and evaluate the course. IKT is defined here as “*the meaningful engagement of the right research users at the right time throughout the project*” [18]. The IKT Guiding Principles (www.iktprinciples.com) guided our collaborative activities (Appendix 3 provides further details).

### Part 1: co-development of the e-learning course

The e-learning course was developed following a systematic, multi-step, theory-informed approach. The development process of the e-learning course targeted the following two activities of the TEL-framework: conduct needs analysis and environmental scan, and document processes, decisions, and final product.

The development of the e-learning course was informed by the following data sources:

- Scientific evidence on effective SCI physical activity counselling and behavior change theories;
- Structured discussions with the multidisciplinary expert panel (*n* = 18);
- Survey data from potential end-users (*n* = 130) about their barriers, needs and preferences regarding training on the best practices;
- Interview data from clients with SCI (*n* = 8) on their perceptions and preferences about receiving physical activity counselling.

**Fig. 1:**
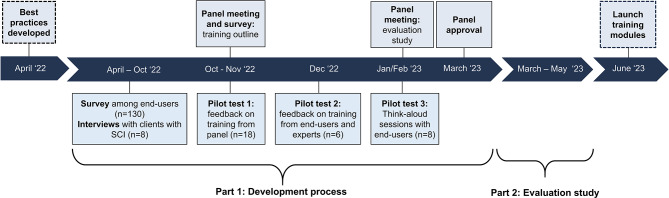
Project overview of the systematic, multistep approach used to co-develop the e-learning course. The development of the course was informed by input and feedback from a diverse group of end-users and experts via online surveys and (think-aloud) interviews. SCI = spinal cord injury. Appendix 4 describes further details on each step of the development process

Throughout the development process we conducted several rounds of pilot testing with panel members (*n* = 18), potential end-users and external experts (*n* = 6). Finally, we conducted think-aloud interviews with another group of potential end-users (*n* = 8) to test the design, usability and content of the e-learning course [19]. Figure 1 summarizes the development process of the course. Additional information on the data procedures and analyses of the steps in the development process are described in Appendix 4 available on the Open Science Framework (OSF) page: https://osf.io/mqxru/.

### Part 2: evaluation of the e-learning course

The evaluation study targeted the following activities of the TEL-framework: assess learning outcomes, test usability, and assess participants’ experience and satisfaction levels.

#### Design and study procedures

**Fig. 2:**
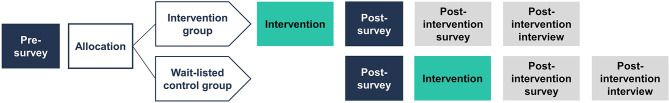
Design of the evaluation study. Participants in the intervention group completed the e-learning course immediately after the baseline survey (pre-survey). Participants in the wait-list control group received access after they completed the one-week follow-up survey (post-survey)

A randomized controlled trial, using a two-group pre-test post-test design, was used to evaluate the effectiveness of the e-learning course. Figure 2 provides an overview of the design of the evaluation study. A sequential explanatory mixed methods design, in which the quantitative (survey) data collection phase was followed by the qualitative (interview) data collection phase, was used to evaluate usability and assess participants’ experiences and satisfaction levels. Interview data were used to confirm and explain the survey findings. Participants were matched by their experiences with working with people with SCI and their level of experience in providing physical activity counselling, and randomly assigned to an intervention group (i.e., completing the e-learning course) or wait-list control group, using a 1:1 allocation ratio. Randomization was done by the research assistant (LK) using a clinical randomization tool [20]. A file was generated by this tool including the allocation sequences for the following four strata: (1) low SCI counselling experiences and low physical activity counselling experiences; (2) high SCI counselling experiences and low physical activity counselling experiences; (3) low SCI counselling experiences and high physical activity counselling experiences; (4) high SCI counselling experiences and high physical activity counselling experiences. Low SCI counselling experience was defined as anyone who supported or counselled 0–4 adults with SCI in the past five years. High SCI counselling experience was defined as anyone who supported or counselled 5 or more adults with SCI in the past 5 years. Low physical activity counselling experience was defined as anyone who had less than 1 year of experience in providing physical activity counselling. High physical activity counselling experience was defined as anyone who had 1 or more years of experience in providing physical activity counselling. Enrollment of participants was done by the first author (FH). LK assigned participants to the intervention group or wait-listed control.

Participants in the intervention group were asked to complete the e-learning course (intervention) within one week after the baseline survey (pre-survey). Immediately after completion of the course, participants in the intervention group were asked to complete two follow-up surveys (post-survey and post-intervention survey) and take part in a 30-minute interview session. Participants in the control group were asked to complete the post-survey one week after completing their baseline survey (pre-survey). After completing the post-survey, participants in the wait-list control group received access to the e-learning course and were invited to complete the course within one week. After completing the course, the control group was invited to complete the post-intervention survey and take part in the 30-minute interview session. Completing the surveys took between 10 and 15 min per survey. All surveys were administrated using Qualtrics XM survey system. The 30-minute online interview sessions were conducted by the first author (FH) via UBC’s Zoom software. Participants received a $50 (CAD) gift card, or an equivalent amount in another currency, for completing all parts of the evaluation study.

The pre-survey and post-survey were used to assess participants’ knowledge and self-efficacy for using the best practices of SCI physical activity counselling. The post-intervention survey and interview data were used to assess usability, participants’ learning experiences and satisfaction levels, and the feasibility of the e-learning course. The interviews were also used as a fidelity check to confirm that participants completed all parts of the e-learning course. Copies of each survey and the interview guide are available on OSF.

#### Participants

Participant inclusion criteria were:

- Work or volunteer as an exercise/lifestyle counsellor, SCI peer mentor, occupational therapist, therapeutic recreation professional, physiotherapist, psychomotor therapist, social worker, kinesiologist, rehabilitation assistant, SCI caregiver, fitness trainer or coach;
- Work or volunteer in Canada, United Kingdom, Ireland, United States of America, Australia, or New Zealand;
- Plan to provide professional guidance or counselling to one or more clients in the next 12 months on starting and/or maintaining a physically active lifestyle. This can include guidance or support as part of the SCI peer mentorship program;
- Are 18 years or older;
- Can read and understand English.

Participants could not take part if they provided any type of feedback on previous versions of the training modules. Participants were recruited via the personal networks of panel members and via social media/ newsletters of community organizations and professional associations. We also invited participants who completed the survey on needs and preferences and provided consent to be contacted again.

#### Power calculation

Based on findings from the evaluation study of a physical activity counselling training toolkit (i.e., ProACTIVE SCI trial) among physiotherapists, very large effect sizes for tested knowledge and confidence levels are expected (Cohen’s *d* = 1.1–2.57) [21]. Based on a sample size calculation (alpha = 0.05 and power = 0.80), a total sample size of 12 would be needed to achieve similar effects. Compared to the ProACTIVE SCI trial, our sample of counsellors was expected to be more diverse in terms of participants’ backgrounds (e.g., physiotherapists, recreation therapists, occupational therapists, SCI peer mentors, health/lifestyle counsellors, university students), level of counselling experience and working country (e.g., Canada, USA, UK). Therefore, a total of 40 participants were recruited in order to take into account the differences in study sample.

#### Measures

##### Primary outcome measures

Our primary outcome measures were: (1) knowledge of the SCI physical activity counselling best practices, and (2) participants’ self-efficacy for using the best practices in conversations about physical activity. Knowledge of the best practices was measured using 15 true/false statements and 3 multiple-choice questions. One point was given for each correct answer. A total sum-score was calculated for all correct knowledge questions (range: 0–18). Self-efficacy was measured using 10 items in which participants were asked to rate their confidence level on a scale from 0 to 10 (0 = not confident I can do at all; 10 = highly confident I can). The average self-efficacy score was calculated for each participant and used for further analyses. The items were constructed using Bandura’s guidance for self-efficacy questionnaires [22]. Knowledge and self-efficacy outcomes were measured at the pre- and post-surveys.

##### Secondary outcome measures

The secondary outcome measures included: (1) usability of the course, (2) learning experiences and satisfaction, (3) feasibility of the course, and (4) capability, opportunity and motivation for using the best practices.

Usability of the e-learning course was assessed using the 10-item System Usability Scale (SUS), a reliable and frequently used questionnaire to measure usability of a variety of technological products, such as websites, mobile applications and e-learning courses [23, 24]. The SUS has widely been used to assess perceived usability of educational technology [25], including e-learning modules for health professionals [26]. The SUS questionnaire provides an overall usability score between 0 and 100, in which a score of 68 or higher is considered as an above average usability [23, 24].

Learning experiences and satisfaction were measured using a selection of items from the learning and satisfaction questionnaire developed by Grieve (2022) [27] in the context of an e-learning course for diabetes prevention coaches. The questionnaire was informed by the Evaluation of Technology-Enhanced Learning Materials: Learners Perceptions (ETELM-LP) questionnaire [15]. We modified Grieve’s questionnaire by shortening the questionnaire to avoid overlap with the SUS-questionnaire, using a 7-point Likert Scale and by tailoring the items to our project. Our modified questionnaire included 6 items related to ‘user engagement’, 1 item related to ‘technical experience’, and 8 items related to ‘satisfaction’. Items were measured on a 7-point Likert scale, in which 1 represents strongly disagree and 7 represents strongly agree. Cronbach’s alphas were between 0.8 and 0.9 for ‘user engagement’ and ‘satisfaction’, allowing for reporting on aggregated scores for each construct.

Feasibility of the course was measured using a modified 10-item questionnaire to assess the affordability, practicability, effectiveness, acceptability, safety, and equity (APEASE) criteria [21, 28]. Items were measured using a 7-point Likert scale (1 = strongly disagree and 7 = strongly agree). Aggregated scores were calculated for the constructs with multiple items (i.e., affordability, practicability, effectiveness, acceptability).

Capability, opportunity and motivation for using the best practices were measured using 9 items (3 items per construct), measured on a 7-point Likert scale (1 = strongly disagree and 7 = strongly agree). The items were constructed using components of the COM-B model [29] and inspired by Hoekstra et al.’s [30] questionnaire to access capability, opportunity, motivation for disseminating research to a non-academic audience. Items of the questionnaire are independent and have meaning on their own. As such, no aggregated scores were calculated.

#### Data analyses

T-test and chi-squared tests were used to identify any significant differences in baseline measures (demographic information, background and expertise, knowledge test, self-efficacy) between the intervention and control group. ANCOVAs with baseline scores (pre-survey) as covariates were conducted to compare post-survey knowledge and post-survey self-efficacy scores (primary outcome measures) between the intervention and control group. We conducted ANCOVAs as we were interested in whether the intervention group had greater knowledge and self-efficacy than the control group after completing the e-learning course. Descriptive statistics (means, standard deviations, ranges) were calculated for the total study sample (intervention and control group) for all secondary outcome measures. Analyses were performed using IBM SPSS Statistics for Windows, Version 28.0.

A directed content analysis [31] of the interview data was conducted to further explore participants’ learning experiences and opinions on completing the e-learning course. Analyses focused on identifying participants’ perceptions and opinions on things they liked about the course, things that could be improved (feedback and comments), and things that participants learned from the course.

## Results

### Part 1: development of the e-learning course

#### Survey

A group of potential end-users (*n* = 130) completed survey questions on barriers, needs and preferences regarding training in the SCI physical activity counselling best practices. Participants’ background/expertise included physical activity or health counsellor (*n* = 52), physiotherapist (*n* = 25), recreation therapist (*n* = 25), occupational therapist (*n* = 12), SCI peer mentor (*n* = 12), and fitness trainer (*n* = 12). Additional information on participants’ demographics and expertise is available in Table S1 of Appendix 5. While the vast majority of participants (84%) indicated that they want to improve their knowledge and skills on SCI-specific physical activity counselling, participants (83%) also indicated that they face barriers to do so. The most frequently mentioned barriers were: lack of time (63%), lack of reimbursement (38%), and lack of resources (18%). Almost all participants (93%) reported that they would be interested in an online training on the best practices for SCI physical activity counselling. Additional survey findings are described in Appendix 5.

#### Interviews

Eight interviews were conducted with clients with SCI who received counselling support. The participants were predominantly Canadian residents, white and heterosexual. Four participants identified as woman (50%). The mean age was 48 ± 10 years old. The interviews lasted on average 41 ± 14 min. Participants talked about various aspects that contributed to positive physical activity counselling support. According to participants, counsellors should be flexible and open minded, knowledgeable, good communicators, and able to create a safe counselling space. Additional findings from these interviews, including quotes from participants, are summarized in Appendix 6.

#### Pilot test 1

Panel members provided written feedback on the first version of the e-learning course. They indicated that the figure of the outline of the course suggested that the best practices should be discussed in a specific order, which was not our intention. Panel members also suggested to focus the course content and language more on physical activity instead of exercise. They mentioned that the word ‘exercise’ may narrow learners’ perceptions on opportunities to engage in physical activity and suggested including more physical activity examples throughout the course. Furthermore, panel members provided specific comments on the clarity of the content throughout the course. Details on how the feedback has been addressed is described in Appendix 7.

#### Pilot tests 2 and 3

A total of 6 external end-users/experts provided written feedback on the second version of the course and 8 participants took part in the think-aloud interviews. Participants (counselor [*n* = 9], researcher [*n* = 8), university student [*n* = 8], SCI peer mentor [*n* = 3], physiotherapist/social worker/fitness trainer [*n* = 4]) were generally positive about the e-learning course. Participants mentioned how the content was relevant and understandable. They also provided editorial suggestions. Most participants liked the design of the e-learning course, but indicated that the banners and videos throughout the course could be more consistent. Some participants raised usability concerns, specifically if the course is to be accessible for people with limited fine motor skills. Additional details on the findings of pilot tests 2 and 3 with a description on how the feedback has been addressed is available in Appendix 7.

The panel decided to co-develop a self-guided e-learning course in which information is presented in different ways (e.g., text, short videos, knowledge quizzes, reflection exercises) to create an engaging learning experience. The panel emphasized to pay specific attention to the inclusivity and accessibility of the course. The pilot testing showed that the course takes on average ~ 2.5 h to complete. The e-learning course is designed to familiarize learners with the best practices for SCI physical activity counselling. The panel formulated the following learning objectives:

- Describe and understand the best practices for SCI physical activity counselling;
- Understand how to use the best practices in conversations about physical activity with adults with SCI;
- Use SCI-specific knowledge on physical activity in conversations with adults with SCI.

**Fig. 3:**
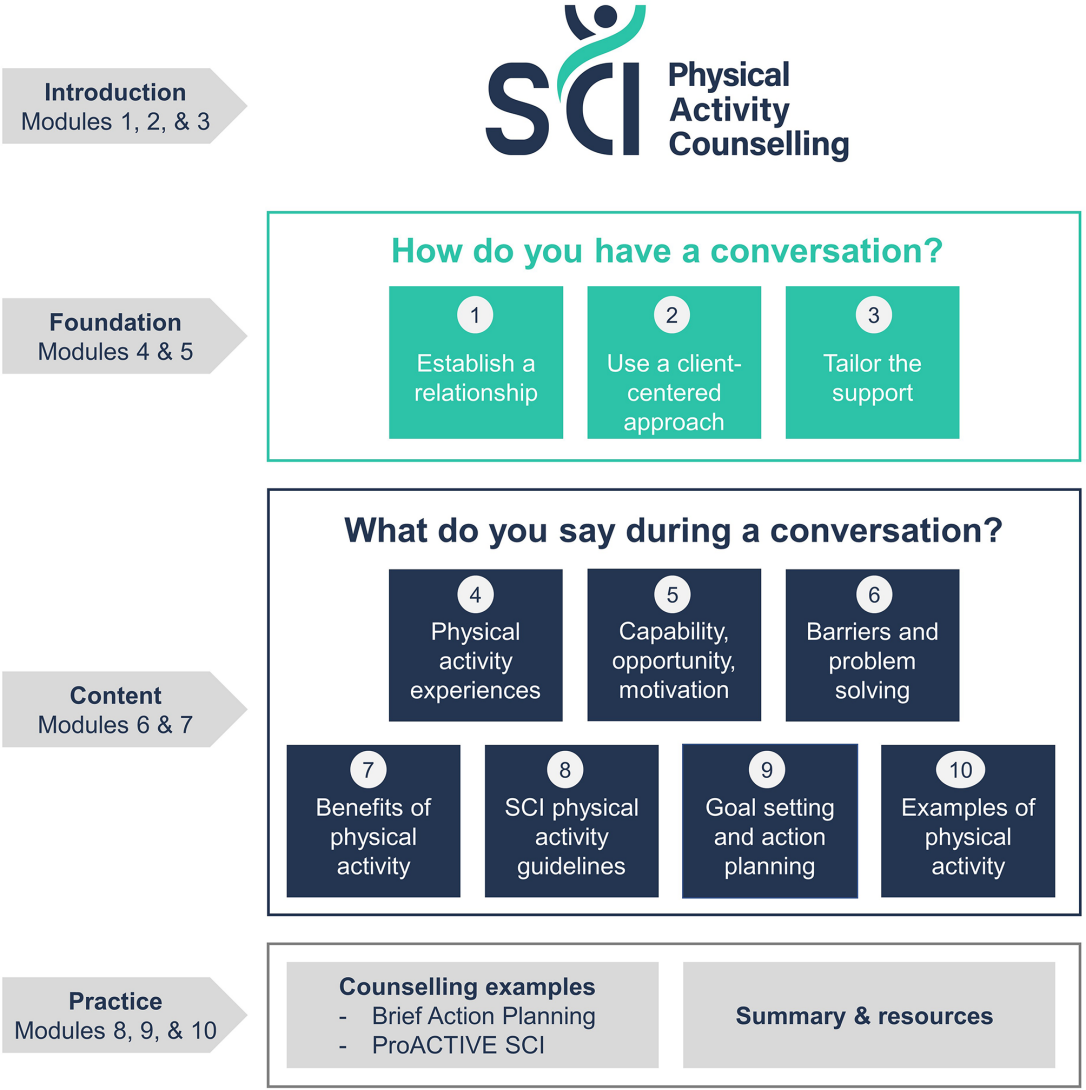
An overview of the content of the e-learning course on SCI physical activity counselling. The course includes 10 modules. Modules 1–3 focus on an introduction to spinal cord injury, physical activity and underlying theoretical approaches. Modules 4–5 focus on the first three best practices on how to have a conversation (best practices 1–3). Modules 6–7 focus on the seven best practices on what to say during a conversation about physical activity with clients with SCI (best practices 4–10). Modules 8–10 provide counselling examples (Brief Action Planning [34], ProACTIVE SCI [21]) and additional resources

The course targets the levels of ‘remembering’ and ‘understanding’ of the revised Bloom’s Taxonomy [32, 33]. The panel decided to co-develop 10 modules explaining each of the best practices, including practical tips and example techniques for using the best practices in physical activity conversations with adults with SCI. Two additional bonus material modules were created with reflection exercises on example counselling sessions. Figure 3 provides an overview of the e-learning course. Appendix 8 provides further details on the course outline, including a description of the didactic methods of the course.

### Part 2: evaluation of the training modules

#### Demographics and expertise

**Fig. 4:**
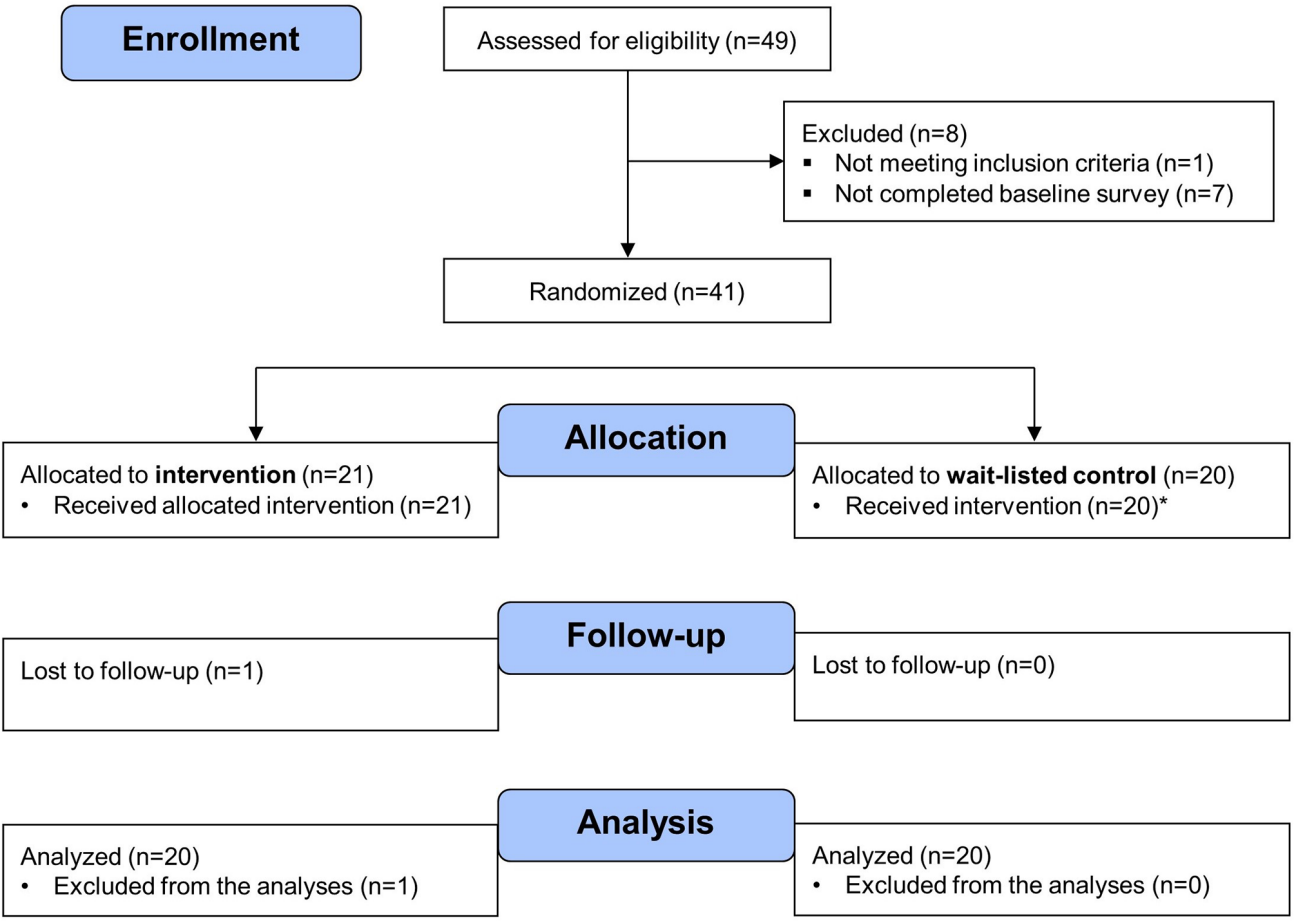
Flow chart of the randomized controlled trial for the analyses of the primary outcome measures. *Participants in the control group received the intervention after the first follow-up survey. A total of 4 participants in the control group did not complete the e-learning course due to concussion (*n* = 1), lack of time (*n* = 1), and unknown reason (*n* = 2). As such, these participants were not included in the analyses of the secondary outcome measures

A total of 41 participants (mean age: 33.9 ± 11.9 years) were enrolled in the randomized controlled trial to evaluate the training between 9 March– 19 May 2023. Data collection took place between 9 March– 1 June 2023. One participant in the intervention group was lost-to-follow-up after starting the intervention and was excluded from the analyses. Figure 4 illustrates the flow chart of participants for the analysis of the primary outcome measures. The majority of enrolled participants identified as a woman (85%) and identified as of white or European descent (81%). Participants’ background and professional expertise included physical activity/health counsellor (27%), physiotherapist (27%), occupational therapist (22%), recreation therapist (17%), fitness trainer (17%), researcher (22%), SCI peer mentor (12%), SCI caregiver (5%), and representative of a community organization (2%). Table 1 provides further details on participants’ demographics and expertise. No significant differences were found in participants’ demographics between the intervention and control group.

**Table 1:** Participants’ demographics and expertise

**Demographics and expertise/background**	**Mean ± SD or n (%)**		
	Intervention group (n = 21)	Control group (n = 20)	Total (n = 41)
Age (years)	33.2 ± 13.1	34.6 ± 10.7	33.9 ± 11.9
Gender identity:			
Man	4 (19%)	1 (5%)	5 (12%)
Woman	17 (81%)	18 (90%)	35 (85%)
Non-binary	-	1 (5%)	1 (2%)
Prefer not to answer	-	-	-
Sexual orientation:			
Straight	18 (86%)	17 (85%)	35 (85%)
Bisexual	-	1 (5%)	1 (2%)
Lesbian	-	1 (5%)	1 (2%)
Pansexual	1 (5%)	-	1 (2%)
Prefer not to answer	2 (10%)	1 (5%)	3 (7%)
Identify as a person with a SCI	2 (10%)	1 (5%)	3 (7%)
Have you ever spent 24 consecutive hours with an individual with a SCI?			
Yes	2 (10%)	7 (35%)	9 (22%)
No	19 (91%)	13 (65%)	32 (78%)
Ethnicity*			
White / European	17 (81%)	16 (80%)	33 (81%)
East/Southeast Asian	2 (10%)	2 (10%)	4 (10%)
Native (First Nations, Métis, Inuk/Inuit)	1 (5%)	1 (5%)	2 (5%)
Latino	-	2 (10%)	2 (5%)
Prefer not to answer	2 (10%)	-	2 (5%)
Background and expertise*			
Physical activity or health counsellor	6 (29%)	5 (25%)	11 (27%)
Physiotherapist	6 (29%)	5 (25%)	11 (27%)
Recreation therapist	4 (19%)	3 (15%)	7 (17%)
Occupational therapist	4 (19%)	5 (25%)	9 (22%)
Fitness trainer/ personal trainer	4 (19%)	3 (15%)	7 (17%)
Researcher	4 (19%)	5 (25%)	9 (22%)
SCI peer mentor	1 (5%)	1 (5%)	2 (5%)
Rehabilitation assistant	2 (10%)	1 (5%)	3 (7%)
Representative of a community organization	-	1 (5%)	1 (2%)
	-	2 (10%)	2 (5%)
Spinal cord injury caregiver Other (kinesiologist, student)	1 (5%)	5 (25%)	6 (15%)
In which country do you currently work and/or volunteer?			
Canada	17 (81%)	14 (70%)	31 (76%)
United States of America	3 (14%)	3 (15%)	6 (15%)
United Kingdom	1 (5%)	3 (15%)	4 (10%)
How many years of experience do you have in providing physical activity counselling?			
None	2 (10%)	1 (5%)	3 (7%)
Less than 1 year	4 (19%)	4 (20%)	8 (20%)
Between 1–3 years	7 (33%)	4 (20%)	11 (27%)
More than 3 years	8 (38%)	11 (55%)	19 (46%)
How many clients with a SCI have you supported or counselled in the past 5 years?			
0	1 (5%)	3 (15%)	4 (10%)
1–4	8 (38%)	5 (25%)	13 (32%)
5–9	4 (19%)	3 (15%)	7 (17%)
10–14	3 (14%)	1 (5%)	4 (10%)
15–24	1 (5%)	-	1 (2%)
> 25	4 (19%)	8 (40%)	12 (29%)
Have you received a formal training or workshop in Motivational Interviewing?			
Yes	6 (29%)	8 (40%)	14 (34%)
No	15 (72%)	12 (60%)	27 (66%)

Notes: *Participants could select multiple answer options

#### Primary outcome measures

The baseline-adjusted ANCOVA analyses on the post-survey knowledge and self-efficacy scores showed that post-knowledge and post-self-efficacy scores were significantly higher in the intervention group compared to the control group (knowledge: *p* <.001; self-efficacy: p = < 0.001; refer to Table 2).

**Table 2:** Participants’ knowledge and self-efficacy scores at baseline (pre-survey) and follow-up (post-survey)

	Pre-survey Mean ± SD (range)		Post-survey Mean ± SD (range)		Baseline-adjusted ANCOVA analyses on post-survey scores			
	Intervention group (*n* = 20)	Control group (*n* = 20)	Intervention group (*n* = 20)	Control group (*n* = 20)	F-value	***p***-value	η^2^	Cohen’s d
Knowledge test* (total score)	10.0 ± 1.9 (7–15)	10.5 ± 1.6 (8–14)	15.5 ± 1.8 (12–18)	11.3 ± 1.5 (8–14)	**72.1**	**< 0.001**	**0.7**	**2.4**
Self-efficacy** (mean score)	6.8 ± 1.9 (2.8–10.0)	6.7 ± 2.0 (3.0–9.2)	8.2 ± 0.7 (6.7–9.2)	6.6 ± 2.1 (2.8–9.5)	**17.7**	**< 0.001**	**0.3**	**1.1**

Notes: *The knowledge test included 18 items/questions. One point was given for each correct answer, indicating that the knowledge test scores could range from 0–18. **Self-efficacy was assessed on a scale from 0–10 (0 = not confident I can do at all; 10 = highly confident I can do). Baseline-adjusted ANCOVAs were conducted to compare post-knowledge and post-self-efficacy scores between intervention and control group

#### Secondary outcome measures

Table 3 summarizes the findings of the usability, learning experiences, satisfaction, and feasibility measures. Appendix 9 provides mean scores for individual items related to all secondary outcome measures, including the capability, opportunity and motivation questionnaire. The average usability system scale score was 80.8 ± 7.9, which is considered as an above average usability [23, 24]. The average user engagement score was 6.1 ± 0.7, indicating that, on average, participants reported that the course was enjoyable and engaging. The average technical experience was 3.0 ± 2.2, indicating that some participants experienced technical difficulties while going through the training. The average satisfaction score was 6.3 ± 0.7, illustrating that overall participants were (very) satisfied with the different components of the e-learning course. Regarding the feasibility of the training, the vast majority of the participants agreed that training is affordable, practical, practicable, effective, acceptable, had no side effects/safety concerns, and was equally beneficial, illustrated by generally high mean scores of the APEASE items (> 6.0 or higher on a 7-point Likert scale). Findings on items related to participants’ motivation for using the best practices were overall positive (means: >6.4 on a 7-point Likert scale; Appendix 9). For example, all participants agreed or strongly agreed with the statement that there is value in using the best practices in a conversation about physical activity with their client with SCI (mean: 6.6 ± 0.5). Findings on items related to capability and opportunity for using the best practices were mixed, but overall positive (means: >5.6 of a 7-point Likert scale; Appendix 9). For example, the majority of participants (60%) agreed or strongly agreed with the statement that they have the resources (e.g., time and money) to use the best practices in a conversation about physical activity with their client with SCI (mean: 5.6 ± 1.1; Appendix 9).

The interview data, collected from 36 participants, confirmed the survey findings that, overall, participants had positive and valuable experiences with completing the e-learning course. Depending on participants’ background and expertise, they took different things (counselling knowledge or skills, confidence) out of the course. While some participants found the SCI-specific information helpful (e.g., SCI Physical Activity Guidelines, SCI-specific physical activity barriers, facilitating action planning), other participants indicated that the information on how to have a conversation (e.g., Ask-Tell-Ask technique, Motivational Interviewing) was more valuable for them. Some participants mentioned that they already applied some of the course content in their sessions with their clients. They said that in particular the modules on how to have a conversation were useful for conversations with all their clients. All participants indicated that they would recommend the e-learning course to others. Participants liked the variation in how information was presented. They also appreciated that the training included a variety of short videos with different presenters, including examples of various counselling sessions. Some participants suggested adding more example videos of counselling sessions, in particular with clients who have no intentions to engage in physical activity. In terms of the inclusivity and accessibility of the course, participants suggested to include closed-captions to the videos. Furthermore, some participants experienced technical issues as the system did not save their progress when they completed parts of the training and came back to the training another day. Appendix 10 summarizes additional information on participants’ learning experiences and perceptions, including suggestions to further improve the e-learning course.

**Table 3:** Usability, learning experiences, satisfaction, feasibility

	Mean ± SD (ranges)
	Total group (*n* = 36)
**Usability**	
System Usability Scale Score*	80.8 ± 7.9 (62.5–90.0)
**Learning experiences**	
User engagement	6.1 ± 0.7 (3.2–7.0)
Technical experience	3.0 ± 2.2 (1.0–7.0)
**Satisfaction**	
Satisfaction levels	6.3 ± 0.7 (4.6–7.0)
**Feasibility (APEASE-criteria)**	
Affordability	6.9 ± 0.3 (6.0–7.0)
Practicability	6.2 ± 0.7 (4.0–7.0)
Effectiveness	6.1 ± 0.7 (4.5–7.0)
Acceptability	6.3 ± 0.7 (4.5–7.0)
Safety	6.9 ± 0.2 (6.0–7.0)
Equity	6.8 ± 0.4 (5.0–7.0)

Notes: *The System Usability Scale Score was calculated using 10-items [23, 24]. Learning experiences were reported using two constructs: ‘user engagement’ and ‘technical experience’. User engagement was assessed with 6 items and technical experience was assessed using 1 item. Satisfaction levels were assessed using 8 items. Affordability, practicability, effectiveness, and acceptability were assessed with 2 items for each construct. Safety and equity were assessed with 1 item per construct. Items related to learning experiences, satisfaction, and feasibility were assessed on 7-point Likert Scale in which 1 = strongly disagree and 7 = strongly agree. The items on affordability, safety and equity were asked during the interview session. Before asking the items on affordability the interviewer explained to the participants that the e-learning course will become freely (no cost) available for everyone after the study has been completed. Appendix 9 provides mean scores for the individual items of the questionnaire, including the Cronbach alphas for each construct

## Discussion

We used a systematic, multi-step, theory-informed approach to co-develop and evaluate an e-learning course on SCI physical activity counselling. The co-development of the course was informed by input and feedback from > 160 end-users and experts. The e-learning course is designed to familiarize learners with the best practices for SCI physical activity counselling [8]. The course was created for anyone who provides professional guidance or counselling to adults with SCI on starting, changing and/or maintaining a physically active lifestyle. The findings of the randomized controlled trial demonstrated improvements in participants’ knowledge on the SCI physical activity counselling best practices and self-efficacy for using the best practices in conversations about physical activity with their clients with SCI. Participants reported above average usability scores, positive learning experiences, and high levels of satisfaction when participating in the course. The overall positive findings of this study demonstrate that the e-learning course is feasible and ready for further implementation in various health and community settings.

This project adds to the existing literature on developing e-learning opportunities for (health) professionals by transparently reporting on a systematic, multi-step, and theory-informed approach to co-develop an evidence-based e-learning course on SCI physical activity counselling. We meaningfully engaged a diverse group of end-users and experts throughout the development process to ensure the course aligns with users’ needs and preferences. Collecting input and feedback from end-users and experts to inform the development process is part of the first activity of the TEL-framework (i.e., conduct needs analysis) [15]. The value and importance of meaningful end-user engagement is consistent with previous projects reporting on the successful development of e-learning opportunities for (health) professionals [27, 35, 36]. However, navigating different opinions and perspectives of end-users and experts added additional complexity to the project and required us to be flexible in our approach. Aligning with the second activity of the TEL-framework [15], we transparently reported on the processes and decisions made throughout the development process. Despite the additional complexity, research showed that meaningful engagement of end-users early and throughout the project can enhance the implementation and relevance of the findings [37, 38].

The findings of the randomized controlled trial demonstrated that participants improved their tested knowledge about the best practices and their self-efficacy for using the best practices after completing the e-learning course. These findings align with previous studies showing increased knowledge and self-efficacy among health, fitness and/or lifestyle professionals after engaging in an (e-learning) training on healthy lifestyle and/or motivational interviewing [27, 39–45]. Within the field of educational psychology, self-efficacy has been identified as an important predictor of students’ motivation to learning [46]. According to Social Cognitive Theory [47], individuals with higher self-efficacy in a specific behavior (i.e., using the SCI physical activity counselling best practices) are more likely to engage in that behavior. As such, the generally high perceived self-efficacy scores (average > 8.0) reported by our participants are promising in terms of the use of the SCI physical activity counselling best practices by professionals across health and community settings. Future research is needed to study whether the e-learning course can contribute to improved physical activity counselling support provided to people with SCI.

The evaluation study demonstrated that participants reported above average usability scores, positive learning experiences, and high satisfaction levels when participating in the e-learning course. The perceived usability score (mean SUS) found in our study (mean: 81) is higher compared to mean SUS scores (ranges: 66–76) reported in a systematic review on usability of different types of educational technology systems (e.g., intervention platforms, university websites) [25]. According to Bangor et al. (2009) [48] a SUS score of 72 represents ‘good’ usability and a SUS score of above 85 represents ‘excellent’ usability. Our e-learning course was rated with a mean SUS score of 81, indicating “good” to “excellent” usability, despite the fact that some participants experienced technical difficulties. Students’ perceived usability of the e-learning course affects their learning experiences as well as their learning outcomes and performance. For example, research showed that higher perceived usability on e-learning platforms of medical education is associated with higher levels of motivation to learn and improved learning outcomes [49]. The high usability scores found in our study align with the positive learning experiences and high satisfaction levels reported by our participants. Furthermore, these overall positive findings on usability, learning experiences and satisfaction levels may be explained by the fact that the course aligns with the UDL guidelines, which emphasize multiple means of representation, action and expression, and engagement [11]. Furthermore, the findings of the feasibility items, following the APEASE criteria, indicated that the training is ready for further implementation in various health and community settings. While APEASE criteria are originally developed in the context of (behaviour change) intervention development [28], we applied the criteria to test feasibility of an e-learning course (intervention). The feasibility of the e-learning course was also confirmed by the interview data, in which participants shared their overall positive e-learning experiences and indicated that they would recommend the course to other people.

### Implications

This project provides a template for reporting on the co-development and evaluation of an evidence-based e-learning resource for a diverse group of health, fitness and lifestyle professionals. Guided by the TEL-framework, we transparently reported on our decision-making processes throughout the development and evaluation phases. We uniquely contributed to the existing literature by using and reporting on our collaborative engagement activities. To illustrate, we used the new IKT Guiding Principles to guide the meaningful engagement of a diverse group of end-users and experts as partners throughout this project [18]. Reporting on our shared principles and related strategies may provide a model for others on ways to meaningfully engage end-users in the development and evaluation of e-learning resources for health, fitness and lifestyle professionals.

Using the feedback and comments from participants in the evaluation study, the e-learning course is being finalized and prepared for further implementation in health and community settings. The e-learning course will be freely available for anyone who is interested. By doing so, a broad group of health, fitness and lifestyle professionals and other individuals can benefit from this SCI physical activity counselling course. Providing a short, evidence-based, e-learning resource can help professionals to better and more effectively support people with SCI to engage in physical activity, and may subsequently contribute to increased physical activity participation in people with SCI.

### Limitations

There are some limitations that should be acknowledged. First, while the e-learning course was created for a broad group of potential end-users from various English-speaking countries, the majority of participants worked in Canada. Furthermore, the development process was informed by input and feedback from end-users and experts from Canada, United States of America and the Netherlands. As such, we do not know to what extent the course resonates with end-users in other English-speaking countries. Furthermore, the vast majority of the study sample and our team identified as white/European, straight, woman without lived experience in SCI. We do not know whether the course will be perceived similarly by end-users from other ethnicities, sexual orientations and/or lived experiences. Further efforts are needed to study whether end-users from other equity-deserving groups will have similar learning experiences and outcomes when engaging in the e-learning course. Second, while we used the TEL-framework as a guide for the development and evaluation processes, we did not use the related ETELM-LP questionnaire [15]. Our team considered the use of this questionnaire, but we decided to use questionnaire items that were shorter and more relevant to our project. The items related to learning experiences and satisfaction were informed by the ETELM-LP. Furthermore, the questionnaire items to assess the feasibility (APEASE), capability, opportunity and motivation measures were modified from previous studies without information on the validity and/or reliability of these questionnaire items. While these survey findings may be interpreted with caution, the interview data confirmed the general positive findings on the course feasibility and participants’ learning experience. Third, we did not compare the e-learning course with other types of training opportunities (e.g., existing online resources, e-learning with interaction options with other learners/educators). Future research is needed to study optimal e-learning methods for a diverse group of health, fitness and lifestyle professionals.

## Conclusion

We used a systematic, multi-step, theory-informed approach to co-develop and evaluate an evidence-based e-learning course on SCI physical activity counselling to support professionals to promote physical activity in their daily practices. The overall positive findings demonstrate that the e-learning course is feasible and ready for further implementation in various health and community settings. The implementation of the e-learning course can help professionals to improve physical activity counselling support that they provide to their clients, and subsequently may contribute to increasing physical activity participation in people with SCI.

### Electronic supplementary material

Below is the link to the electronic supplementary material.

Supplementary Material 1

## Data Availability

The survey datasets generated and analyzed during this project are available in the Open Science Framework (OSF) repository (https://osf.io/mqxru/) Summaries of the findings of the interview data as well as additional materials are published in supplementary files and on OSF.
