# Supplementary material for: The co-development and evaluation of an e-learning course on spinal cord injury physical activity counselling: a randomized controlled trial
Source: BMC Med Educ. 2024 Mar 6;24:240. doi: 10.1186/s12909-024-05141-7 (PMC10916033; doi:10.1186/s12909-024-05141-7)
Supplement: Supplementary file 1 — Supplementary Material 1 [file 12909_2024_5141_MOESM1_ESM.docx]

**Supplementary Files**

**Table of Contents – Supplementary Files**

| **Appendix 1:** | Criteria for Reporting on Development and Evaluation of Professional Training interventions in Healthcare (CRe-DEPTH) |
| --- | --- |
| **Appendix 2:** | Panel members’ names, expertise, and organizations and group-level demographics |
| **Appendix 3:** | Integrated knowledge translation approach: collaborative activities |
| **Appendix 4:** | Methods and procedures of the steps in the development process |
| **Appendix 5:** | Summary of survey findings on counsellors’ needs and preferences |
| **Appendix 6:** | Summary of interview findings with clients with spinal cord injury |
| **Appendix 7:** | Summary of findings from pilot testing |
| **Appendix 8:** | Description of the e-learning course on Spinal Cord Injury Physical Activity Counselling |
| **Appendix 9:** | Results of secondary outcome measures |
| **Appendix 10:** | Summary of findings of interviews on participants’ learning experiences and perceptions |

**Table of Contents – Open Science Framework**

- **Panel meeting materials**
  - Meeting June 2022
  - Meeting October 2023
  - Meeting Feb 2023
- **Data source 3: International survey among counsellors**
  - Consent form: English & Dutch
  - Survey: English and Dutch
- **Data source 4: Interviews with clients with SCI**
  - Consent form
  - Interview guides
- **Pilot tests 1 - 3**
  - Think-aloud protocol
- **Evaluation study**
  - Surveys
  - Interview guide
  - Survey data
- **E-learning course**
  - List of references and resources

*Link to Open Science Framework page:* [*https://osf.io/mqxru/*](https://osf.io/mqxru/)

**Appendix 1:** Criteria for Reporting on Development and Evaluation of Professional Training interventions in Healthcare (CRe-DEPTH)

**Table S1:** Criteria for Reporting on Development and Evaluation of Professional Training interventions in Healthcare

| **Criterion** | **Description** |
| --- | --- |
| ***Development of the training*** | |
| Item 1: Description of the aim or objectives of the training | Main text – methods section |
| Item 2: Description of the underlying theoretical framework | Main text – introduction and methods section |
| Item 3: Description of the developmental process | Main text – methods section and appendices and OSF. |
| Item 4: Description of target population and setting of the training | Main text – introduction and methods section |
| Item 5: Description of the educational resources | Main text – results and Appendix 8 |
| ***Characteristics of the training*** | |
| Item 6: Description of the content of the training | Main text – Figure 3 and Appendix 8 |
| Item 7: Description of the format | Main text – results (online) |
| Item 8: Description of the didactic methods of training | Appendix 8 |
| Item 9: Description of tailoring of the training | Appendix 8 |
| ***Characteristics of the providers/trainers*** | |
| Item 10: Description of the providers of the training | Appendices 2-3 |
| ***Assessment of the training outcomes*** | |
| Item 11: Description of the measured outcomes | Main text – methods |
| Item 12: Description of the applied assessment method, including validity and reliability | Main text – methods and discussion |

**Appendix 2:** Panel members’ names, expertise, and organizations and group-level demographics

The current project is part of a larger project on understanding and improving spinal cord injury (SCI) physical activity counselling. The first part of the project included the co-development of best practices for SCI physical activity counselling^[[1]](#footnote-1)^. The second part of the project included the co-development and evaluation of an e-learning course on these best practices. A diverse expert panel including 15 members (counsellors, researchers, people with SCI) was established to co-develop the best practices. For the current project on the co-development and evaluation of the e-learning course, we expanded the existing expert panel with 3 additional members: an educational consultant for inclusion (EE), a physiotherapist working in Canada (EP), and an adaptive fitness coach and educator (MW). Table 2.1 provides an overview of panel members’ names, expertise and organizations and Table 2.2 provides an overview of group-level demographics. Please refer to Hoekstra et al. (2023) for additional details on the panel selection and considerations.

**Table S2.** Panel members’ names, expertise, and organizations (n=18)

| Names | Organization | Country | Expertise / background |
| --- | --- | --- | --- |
| Anniek van Vilsteren | Vogellanden | NL | Counsellor + Psychomotor therapist |
| Delaney Collins | UBC / Dalhousie University | Canada | Counsellor + Occupational therapy student |
| Diane Rakiecki | UBC | Canada | Physical education (BPE) background + lived experience in SCI + counsellor |
| Electra Eleftheriadou | UBC | Canada | Educational Consultant for Inclusion |
| Emily Giroux | UBC | Canada | Counsellor + trainer + researcher + partner of someone with SCI |
| Erica de Passillé | Horizon Health Network | Canada | Physiotherapist |
| Femke Hoekstra | UBC | Canada & NL | Researcher |
| Heather Gainforth | UBC | Canada | Researcher |
| Jasmin Ma | UBC | Canada | Researcher + kinesiologist + counsellor + trainer |
| Jereme Wilroy | University of Alabama at Birmingham | USA | Health coach + lived experience in SCI + researcher |
| Kathleen Martin Ginis | UBC | Canada | Researcher |
| Megan Williamson | Ocean Rehab and Fitness | Canada | Adaptive fitness coach + educator |
| Rita van den Berg-Emons | Erasmus MC | NL | Researcher |
| Rogier Broeksteeg | Rijndam Revalidatie | NL | Counsellor + health coach + physiotherapist |
| Shannon McCallum | Therapeutic Recreation Program | Canada | Recreation therapist + professor |
| Shannon Rockall | Praxis Spinal Cord Institute | Canada | Occupational therapist |
| Sonja Gaudet | UBC + SCI BC | Canada | SCI peer mentor + counsellor + lived experience in SCI |
| Stephanie Corras | Get in Motion coordinator | Canada | Counselling services + training counsellors |

*Notes:* Laura Kuipers was involved in the project as trainee and research assistant.

**Table S3**: Panel members’ demographics

| **Demographics** | **Mean ± SD or n (%)** |
| --- | --- |
| **Age (years)** | 39.4 ± 36.0 |
| **Gender identity:**  Woman  Man | 15 (88.2%)  2 (11.8%) |
| **Sexual orientation:**  Straight  Prefer not to answer | 15 (88.2%)  2 (11.8%) |
| **Identify as a person with a SCI** | 3 (17.6%) |
| **Identify as a person with a disability** | 3 (17.6%) |
| **Ethnicity**  White (European descent)  East/Southeast Asian  First nation  Middle-Eastern | 16 (94.1%)  2 (11.8%)  1 (5.9%)  1 (5.9%) |

*Notes:* Data based on information collected from 17 panel members.

**Appendix 3**: Integrated knowledge translation approach: collaborative activities

IKT is defined here as the “*meaningful engagement of the right research users at the right time throughout the SCI research process*.”

The 8 IKT Guiding Principles ([www.IKTprinciples.com](http://www.IKTprinciples.com)) used to guide our approach include:

- Partners develop and maintain relationships based on trust, respect, dignity, and transparency.
- Partners share in decision-making.
- Partners foster open, honest, and responsive communication.
- Partners recognize, value, and share their diverse expertise and knowledge.
- Partners are flexible and receptive in tailoring the research approach to match the aims and context of the project.
- Partners can meaningfully benefit by participating in the partnership.
- Partners address ethical considerations.
- Partners respect the practical considerations and financial constraints of all partners.

**Table S4:** provides an overview of the key strategies used to engage our panel members in our project.

| **Research phase** | **Strategy / Activity** | **When** | **Comments** |
| --- | --- | --- | --- |
| *Planning* | Panel members were not involved in grant writing process. | July 2020 |  |
| *Conduct* | **Online meeting with panel** to discuss potential strategies and activities that could/should/shouldn’t be used when providing physical activity counseling for adults with spinal cord injury. | October 2021 | Meeting materials are available on OSF.  Meeting has been recorded. |
| *Conduct* | **Online meeting with panel** to discuss survey findings and outline of the training modules | June 2022 | Meeting materials (agenda, slides, minutes) are available on OSF.  Meeting has been recorded. |
| *Conduct* | **Online survey with panel** to provide feedback/ comments on outline of the training and learning objectives and to ask how members would like to be involved. | July/August 2022 | Survey materials are available on OSF. |
| *Conduct* | **Written feedback on the first draft of the training modules.** The first author (FH) circulated the training modules and panel members provided written feedback. | October 2022 | First draft of the training module is available on OSF.  A summary of the feedback is also available on OSF. |
| *Conduct* | **Online meeting with panel** to discuss first draft of the training modules using the following guiding questions:   - What do you like about the training? - What do you think should be improved? - What content are you missing? | October 2022 | Meeting materials (agenda, presentation slides) are available on OSF.  Meeting has been recorded. |
| *Conduct* | **Written feedback** by individual panel members on (parts of) the training. | Dec 2022 – Jan 2023 | A summary of the feedback is available on OSF. |
| *Conduct* | **Online panel meeting** to discuss progress of training modules and design of evaluation study. | Feb 2023 | Meeting materials are available on OSF. |
| *Conduct* | First author (FH) **emailed** panel members the version of the training modules to be approved for the evaluation study. | Feb 2023 | The draft of the training modules is available on OSF. |
| *Conduct* | Panel members **reviewed** the methods / results section of the manuscript and provided high-level feedback. | May 2023 | Minor suggestions / feedback were provided. |
| *Conduct* | Panel members **reviewed** the full manuscript and provided feedback. | May 2023 | Minor suggestions / feedback were provided. |
| *Conduct* | Panel members **approved** the manuscript | June 2023 |  |

Notes: OSF = Open Science Framework

**Appendix 4:** Methods and procedures of the steps in the development process

The development of the e-learning course was informed by the following data sources:

1. **Data source 1**: Scientific evidence on effective SCI physical activity counselling and behavior change theories;
2. **Data source 2**: Structured discussions with the multidisciplinary expert panel
3. **Data source 3:** Survey data from potential end-users (n=130) about their barriers, needs and preferences regarding training on the best practices
4. **Data source 4:** Interview data from clients with SCI (n=8) on their perceptions and preferences about receiving physical activity counselling.

The following rounds of pilot testing were conducted:

1. **Pilot test 1:** Written feedback from panel members
2. **Pilot test 2:** Written feedback from external end-users and experts
3. **Pilot test 3:** Think-aloud interview sessions with external end-users

**Data source 1 + 2: Scientific evidence and structured discussions**

Details on the scientific evidence are described in Hoekstra et al. (2023). Details on structured discussions on described in Appendix 2 and OSF.

**Data source 3: An international survey on SCI physical activity counselling**

We conducted an online international survey to identify counsellors’ barriers, needs and preferences regarding providing and improving SCI-specific physical activity counselling. We invited exercise/lifestyle counsellors, SCI peer mentors and a variety of professionals who are planning to provide professional guidance or counselling to one of more clients in the next 12 months on starting and/or maintain a physically active lifestyle.

To be eligible to participate in this study, participants must:

1. work or volunteer as an exercise/lifestyle counsellor, SCI peer mentor, occupational therapist, therapeutic recreation professional (e.g., recreation therapist/specialist), physiotherapist, psychomotor therapist, social worker, kinesiologist, SCI caregiver, fitness trainer or coach; AND
2. work or volunteer in Canada, United Kingdom, Ireland, United States of America, Australia, New Zealand or the Netherlands; AND
3. are planning to provide professional guidance or counselling to one or more clients in the next 12 months on starting and/or maintaining a physically active lifestyle. This can include guidance or support as part of the SCI peer mentorship program; AND
4. are over the age of 18; AND
5. can read and understand English.

The study involved a 20-minute online survey. The survey includes questions related to:

- Counselling experience
- Knowledge, skills and confidence on SCI exercise counselling
- Barriers to improving SCI exercise counselling
- Needs and preferences on improving SCI-specific exercise counselling
- Demographic information.

The English and Dutch version of the surveys are available on OSF. Further details on methods and procedures are described in Kuipers et al. (2023).

**Data source 4: Interview data from clients with SCI**

Online interviews were conducted to explore experiences, perceptions, and preferences of adults with spinal cord injury (SCI) who received physical activity counselling.

To be eligible to participate in this study, participants must:

- have received professional guidance or counselling in the past 12 months on starting, changing and/or maintaining a physically active lifestyle. This can include guidance or support from an occupational therapist, recreation therapist, physiotherapist or SCI peer mentor; AND
- have been diagnosed with a spinal cord injury more than 1 year ago; AND
- are a resident of Canada or United States of America; AND
- are over the age of 18; AND
- can read and understand English.

Participants’ demographic information was collected via short online survey. The interview session included questions about participants’ experiences, perceptions and preferences about receiving exercise counseling. A copy of the survey and the interview guide is available on OSF.

**Pilot test 1: Written feedback from panel**

Panel members were invited to review the training modules and provide written feedback. Participants reviewed the training modules via Articulate Rise review platform. They could either provide feedback within the review platform or via a separate document. Feedback was sent to the first author (FH).

**Pilot test 2: Written feedback from external end-users and experts**

Potential external end-users (e.g., lifestyle counsellors, physiotherapists, university students’ kinesiologists etc.) were invited to review the training modules and provide written feedback. Participants reviewed the training modules via Articulate Rise review platform. They could provide feedback within the review platform or via a separate document that was sent to the first author (FH).

**Pilot test 3: Think-aloud interview session with external end-users**

Think-aloud interviews were conducted to explore end-user’s response to the design, content and usability of the SCI physical activity counselling training modules. The think-aloud interviews were conducted using a structured interview guide in which the participant was asked to complete parts of the training modules while verbalizing their thoughts and opinions. Prior to the interview sessions, participants were asked to review the outline of the training modules. The duration of the interview session varied, with a maximum of 2.5 hours, including one or two small breaks (if needed). Additional details on the think aloud interview procedures including the interview guide are available on OSF.

**Appendix 5:** Summary of survey findings on counsellors’ barriers, needs and preferences of providing and improving spinal cord injury physical activity counselling

A total of 130 potential end-users participated in the survey study on counsellors’ barriers, needs and preferences, of which 75 participated in the English survey and 55 in the Dutch survey. A copy of the surveys (English and Dutch) including the consent form with additional information is available on Open Science Framework (OSF). Table S5 provides an overview participants’ demographics and expertise. Table S6 provides key findings on participants’ barriers, needs and preferences.

**Table S5:** Participants’ demographics and expertise/background

| **Demographics and expertise/background** | **Mean ± SD or n (%)** | |
| --- | --- | --- |
|  | **English**  **survey**  **(n=75)** | **Dutch**  **survey**  **(n=55)** |
| Age (years) | 34.4 ± 10.5 | 37.5 ± 10.9 |
| Gender identity:  Man  Woman  Prefer not to answer | 12 (16%)  55 (73%)  4 (6%) | 13 (28%)  33 (72%)  - |
| Sexual orientation:  Straight  Bisexual  Gay  Lesbian  Not listed  Prefer not to answer | 59 (79%)  2 (3%)  1 (1%)  1 (1%)  1 (1%)  7 (95%) | 36 (78%)  -  1 (2%)  -  -  8 (15%) |
| Identify as a person with a SCI | 7 (9%) | 2 (4%) |
| Have you ever spent 24 consecutive hours with an individual with a SCI?  Yes  No | 19 (25%)  52 (69%) | 10 (22%)  36 (78%) |
| Ethnicity*  Black  East/Southeast Asian  Latino  Native (First Nations, Métis, Inuk/Inuit)  Middle-Eastern  South Asian  White / European  Prefer not to answer | 1 (1%)  2 (3%)  -  2 (3%)  1 (1%)  2  58 (77%)  6 (8%) |  |
| In which country were you born?  Netherlands  Other country (Greece, Indonesia, Austria) |  | 52 (95%)  3 (6%) |
| Background and expertise  Physical activity or health counselor  Physiotherapist  Recreation therapist  Occupational therapist  Fitness training/ personal trainer  SCI peer mentor  Other coach | 22 (29%)  15 (20%)  22 (29%)  6 (8%)  9 (12%)  10 (13%)  25 (33%) | 30 (55%)  13 (24%)  3 (6%)  6 (11%)  3 (6%)  2 (4%)  14 (26%) |
| In which country to do you live?  Canada  United States of America  United Kingdom | 58 (77%)  9 (12%)  4 (5%) |  |
| How many years of experiences do you have in providing physical activity counselling?  None  Less than 1 year  Between 1-3 years  More than 3 years | 2 (3%)  8 (11%)  13 (17%)  52 (69%) | 4 (7%)  8 (15%)  15 (28%)  27 (50%) |
| How many clients with a SCI have you supported or counselled in the past 5 years?  0  1-4  5-9  10-14  15-24  >25 | 2 (3%)  30 (40%)  4 (5%)  11 (15%)  9 (12%)  19 (25%) | 17 (32%)  20 (37%)  3 (15%)  -  3 (6%)  3 (6%) |

Notes: *The item on ethnicity was included in the English survey only.

**Table S6:** Participants’ barriers, needs and preferences related to providing and improving physical activity counselling.

|  | **Mean ± SD or n (%)** | |
| --- | --- | --- |
| **Survey items** | **English**  **Survey**  **(n=75)** | **Dutch**  **Survey**  **(n=55)** |
| ***Barriers*** |  |  |
| Are you experiencing any barriers to improving your knowledge and skills on SCI-specific physical activity counselling?  No  Lack of time  Lack of reimbursement  Disbelief that it will change my knowledge/skills  Lack of resources  Lack of interest  Feeling it would not be beneficial for me  Other | 9 (13%)  48 (67%)  34 (47%)  1 (1%)  15 (21%)  3 (4%)  2 (3%)  10 (14%) | 8 (17%)  27 (57%)  11 (23%)  1 (2%)  6 (13%)  3 (6%)  2 (4%)  15 (32%) |
| Are you experiencing any barriers to improving your confidence in delivering physical activity counselling to adults with SCI?  No  Lack of practice  Lack of knowledge  Lack of skills  Disbelief that it will change my confidence level  Feeling it would not be beneficial for me  Other | 25 (34%)  34 (47%)  28 (39%)  17 (24%)  2 (3%)  1 (1%)  7 (10%) | 20 (43%)  16 (34%)  14 (30%)  9 (19%)  1 (2%)  1 (2%)  3 (6%) |
| ***Needs and preferences*** |  |  |
| I want to improve my knowledge and skills on SCI-specific physical activity counselling | 6.1 ± 0.9  (2.0 – 7.0) | 5.1 ± 1.6  (1.0 – 7.0) |
| I am interested to take part in a training/workshop on best practices for SCI-specific physical activity counselling. | 6.0 ± 0.9  (1.0 – 7.0) | 5.0 ± 1.6  (1.0 – 7.0) |
| I think there is a value in taking part in a training/workshop on best practices for SCI-specific physical activity counselling | 6.3 ± 0.9  (4.0 – 7.0) | 5.7 ± 1.2  (1.0 – 7.0) |
| I am motivated to take part in a training/workshop on best practices for SCI-specific physical activity counselling. | 6.0 ± 1.0  (2.0 – 7.0) | 5.1 ± 1.6  (1.0 – 7.0) |
| Would you use an online resource/toolkit outlining the evidence-based best practices for SCI physical activity counselling?  Yes  No | 72 (100%)  0 | 39 (83%)  8 (17%) |
| Would you be interested in attending one or more online training session(s) or webinars about the new evidence-based best practices for SCI physical activity counselling?  Yes  No | 69 (97%)  2 (3%) | 37 (80%)  9 (16%) |
| Would you be interested in online training modules that are free of charge to teach counsellors the best practices for SCI physical activity counselling?  Yes  No | 71 (100%)  0 | 39 (85%)  7 (15%) |
| What aspects would you like to see in a training on SCI physical activity counselling?  Short videos on the content  Quizzes to test my knowledge  Links to other resources/literature  Example videos of a ‘good’ counselling session  Practice/role play a counselling session with other participants  Practice/role play a counselling session with actors  Opportunities to receive feedback on my counselling skills  Discussion forum to post questions and share experiences with other counsellors  Other | 64 (85%)  37 (49%)  47 (63%)  54 (72%)  28 (37%)  21 (28%)  33 (44%)  34 (45%)  5 (7%) | 33 (60%)  21 (38%)  24 (44%)  36 (66%)  7 (13%)  10 (18%)  20 (36%)  17 (31%)  5 (9%) |
| What topics related to SCI physical activity counselling would you like to learn more about?  Building rapport with your client  Motivational interviewing  Physical activity barriers in adults with SCI  Goal setting and action planning  Tailoring the support  Assessing clients’ physical activity levels  Benefits of physical activity for adults with SCI  SCI physical activity guidelines | 25 (33%)  40 (53%)  45 (60%)  34 (45%)  38 (51%)  48 (64%)  28 (37%)  37 (49%) | 4 (7%)  14 (26%)  34 (62%)  18 (33%)  23 (42%)  22 (40%)  12 (22%)  21 (38%) |
| What types of resources or tools on SCI physical activity counselling are you interested in?  Example counselling questions/ flow chart  Videos with example counselling sessions  Action plan scheme  List of behaviour change techniques | 54 (72%)  49 (65%)  52 (69%)  54 (72%) | 22 (40%)  24 (44%)  28 (51%)  26 (47%) |
| What would be the ideal total length to complete all training modules on SCI physical activity counselling?  Less than 4 hours  4 – 8 hours  8 – 12 hours  More than 12 hours | 29 (39%)  29 (39%)  10 (13%)  2 (3%) | 22 (49%)  14 (31%)  9 (20%)  - |

*Note:* The total number of participants that completed each item varies due to missing values.

**Appendix 6:** Summary of interview findings with clients with spinal cord injury

Eight interviews were conducted with clients with spinal cord injury (SCI) who received physical activity counselling. Interviews were conducted via Zoom and lasted on average 41 ± 14 minutes. The participants were predominantly Canadian residents, white and heterosexual. The mean age was 48 ± 10 years old. Four participants identified as woman (50%). Open Science Framework (OSF) provides additional details on participants’ characteristics, including the interview guide. This appendix provides a summary of participants’ perspectives and opinions on key aspects for good counselling support.

Participants talked about various aspects that contributed to a positive physical activity counselling support. According to participants, counsellors should be flexible and open minded, knowledgeable, good communicators, and able to create a safe counselling space.

**Flexibility and open mindedness**. Some participants greatly appreciated their counsellor's ability to adapt and to be flexible, while others expressed that they wished their counsellor had displayed more of this quality. Flexibly is required, as clients’ needs can vary each session. Participants mentioned that counsellors should be flexible, creative, and be able to think outside the box. Some participants said that counsellors should avoid going by the book too much and instead try multiple approaches while leaving room for a client's capabilities and wishes. For example, Robert talked about the importance of tailoring the counselling to each client.

Robert: ‘*They've tailored it to everybody so everybody can participate. That's probably the most wonderful thing about it, that it's, you know, not one shoe fits all*.’

Furthermore, participants highlighted that counsellors should be open-minded about the possibilities for their client, as it often hard to predict how much a person with SCI can do.

**Knowledge and education.** Counsellor’s knowledge about physical activity and SCI is another important aspect that contributes to a positive counselling experience. According to the participants, different types of knowledge are important to feel confident in their counsellors' abilities, to feel recognized and understood, and to provide informational support. For example, knowledge obtained from an academic education such as neurological and physiological knowledge, knowledge from work experiences with a specific target group such as working with people with SCI, knowledge from lived experience and/or specific knowledge about the abilities and limits of their client's body. Participants were also able to trust in the competence of their counsellors even when they did not necessarily possess specialized knowledge in SCI or had experiences with working with people with SCI, as long as the counsellor showed willingness to learn more and educate themselves. For example, Jessica talked about how her counsellor, who did not have a lot of experience with working with people with SCI, educated themselves and showed their willingness to learn.

Jessica: *“I kept going back to her even though spinal cord injury is not like specifically her specialty, but she is willing to learn. So it was good.”.*

**Communication skills**. The participants also highlighted the need for good communication with their counsellor, which helped to build a trusty and meaningful relationship between counsellor and client. According to the participants, optimal communication is not one-sided, but a mutual effort from both the client and the counsellor. To illustrate, counsellors should have good listening skills to understand their clients’ goals, barriers, needs and wishes. Participants mentioned that it is important that clients should feel engaged, can ask questions during the counselling sessions, and take ownership of their progress. They also said that the counsellor should avoid using jargon and speaking too quickly.

**Ability to create a safe counselling space.** Participants mentioned that there should be a safe and pleasant atmosphere during physical activity counselling. Most participants experienced that their counsellor was friendly, non-judgmental, considerate and compassionate, which helped to feel them comfortable and safe throughout the counselling. Michael however, had the opposite experience. He felt that his counsellor was not very patient, lacked understanding or judged him by his performance or progress. This left him feeling blamed, frustrated, or like a disappointment whenever he failed or was not making progress. If physical activity counselling was provided in a group setting, not only the counsellor was responsible for creating a safe space, but also the participants and their peers.

**Appendix 7**: Summary of findings from pilot testing

This appendix provides a summary of the key findings (feedback and comments) of each round of pilot testing, including a short description on how we addressed the feedback.

**Pilot test 1: Written feedback from panel**

Panel members provided written feedback on the first version of the training modules. This version did not include videos. The key points of feedback and how they have been addressed were:

- Figure of the outline of the training should be revised*.* Panel members suggested to change the figure as the figure looked like a flow chart, which could suggest that the best practices should be discussed in a specific order, which was not our intention.
  - To address this point, we revised the figure (refer to Figure 3 in main text). The figure does not look like a flow chart. We included comments throughout the training that the order of discussing the best practices is flexible. We added two modules with example counselling flow charts (Modules 8 + 9 on ProACTIVE SCI and Brief Action Planning) to provide learners examples of how best practices could be discussed.
- More focus on physical activity instead of exercise**.** Panel members suggested focusing more on active lifestyle / physical activity instead of exercise only. They mentioned that the word ‘exercise’ may narrow learners’ perceptions on opportunities to engage in physical activity and suggested including more physical activity examples throughout the course.
  - To address this point, we added an introductory module including terminology and definitions related to physical activity and exercise (Module 2). Physical activity examples are included throughout the course.
- Specific comments throughout on clarity of the content throughout the training. Panel members provides specific feedback and comments on the content and language throughout the training to improve clarity and flow.
  - We made editorial edits throughout the course to improve the clarity and flow of the content, as suggested by the panel.

**Pilot test 2: Written feedback from external end-users and experts on Version 2 of the course**

A total of 6 external end-users/experts provided written feedback on Version 2 of the course. Participants’ demographics are presented in Table S7. Version 2 of the course included content of Modules 1-10, but without the recorded videos. Participants were asked to share what they liked about the training and things that could be improved.

Key feedback and comments from participants included:

- Suggestions to improve structure and flow on how the information was presented**.** Several participants provided suggestions throughout the course to revise the order and flow of the information.
  - We made various editorial and structural changes throughout the course. Some of the comments were also addressed by incorporating the videos in the course.
- Concerns about the usability of the course for people with limited hand function. For example, the matching exercises in which users had to click and drag boxes might be challenging for people with limited hand function.
  - We replaced the knowledge exercises/ quizzes with click/drag functions to type of questions that do not require dragging function (multiple-choice questions).
- Provide examples of counselling sessions.
  - We added example videos of counselling sessions throughout the course. We also added bonus-material with additional example videos and reflection exercises.
- Editorial suggestions and typos. Participants provided various editorial suggestions and pointed out typos and inconsistencies.
  - We made editorial changes throughout the course.

This version of the course was also reviewed by our inclusive education expert, who provided feedback and comments from a pedagogical perspective as well as feedback to improve accessibility and inclusivity of the course. The first author and inclusive education expert discussed the feedback resulting in the following changes to the course:

- Changes to lay-out and fonts to ensure use of high-contrast colours
- Adding reflection exercises to the course to stimulate meaningful learning experiences
- More focus on consistency in the course to minimize that learners will get distracted
- Optimize relevance, value and authenticity of the training to keep the learner’s engagement high

**Pilot test 3: Think-aloud interview session with external end-users on Version 3 of the course**

A total of 8 participants took part in the think-aloud interview sessions. Participants’ demographics are presented in Table S7 together with participants who took part in pilot test 2 and 3. Participants went through 3 – 5 modules per session. Version 3 of the course included Modules 1-10, including all videos as well as Bonus material. Improvements to the course were made throughout the pilot testing, so that new participants would review a revised and improved version of the course. Key feedback and changes to the course were:

- More consistency in how information was presented.
  - We re-organized some content and included more consistency in the use of fonts and banners and colours.
- Number the best practices in Modules 4 – 7 to help structure the content.
  - We numbered the best practices in the modules as well as in the outline figure.
- Including the flashcards at the end of the module as a summary
  - We made edits to the flashcards and presented them at the end of the module as an interactive summary of the content.
- Editorial suggestions and typos.
  - We made editorial changes throughout the course to improve clarity and consistency.
- Adding more quizzes and knowledge checks
  - We added quizzes between modules for additional knowledge checks for participants.
- Adding information on quality participation (not just focus on quantity)
  - We have not yet added this information. We will incorporate this in the next version of the course.

**Table S7:** Participants’ demographics and expertise/background: pilot test 2 and 3

| **Demographics and expertise/background** | **Mean ± SD or n (%)** |
| --- | --- |
|  | **Pilot tests participant**  (n=14) |
| Age (years) | 30.3 ± 11.6 |
| Gender identity:  Man  Woman | 6 (43%)  8 (57%) |
| Sexual orientation:  Straight  Asexual  Bisexual  Gay  Lesbian  Not listed  Prefer not to answer | 10 (71%)  1 (7%)  2 (14%)  -  -  -  1 (7%) |
| Identify as a person with a SCI | 3 (21%) |
| Identify as a person with a disability | 4 (29%) |
| Ethnicity  Black  East/Southeast Asian  Latino  Native (First Nations, Métis, Inuk/Inuit)  Middle-Eastern  South Asian  White / European  Prefer not to answer | 1 (7%)  1 (7%)  2 (14%)  -  1 (7%)  -  11 (79%)  - |
| Background and expertise*  Physical activity or health counselor  Physiotherapist  Recreation therapist  Occupational therapist  Fitness training/ personal trainer  SCI peer mentor  Social worker  Researcher  University student  Other expertise (para-athlete) | 9 (64%)  1 (7%)  -  -  2 (14%)  3 (21%)  1 (7%)  8 (57%)  8 (57%)  1 (7%) |
| Experience with completing other e-learning training on other topics?  Yes  No | 12 (86%)  2 (14%) |

**Appendix 8**: Description of the e-learning course on Spinal Cord Injury Physical Activity Counselling

**Description of the content of the e-learning course**

The e-learning was designed to familiarize learners with the best practices for spinal cord injury (SCI) physical activity counselling to support the use of these best practices in conversations about physical activity with adults with SCI.

After completing the course, learners will be able to:

- Describe and understand the best practices for SCI physical activity counselling.
- Understand how to use the best practices in conversations about physical activity with adults with SCI.
- Use SCI-specific knowledge on physical activity in conversations with adults with SCI.

The course targets the levels of ‘remembering’ and ‘understanding’ of the revised Bloom’s Taxonomy^[[2]](#footnote-2)^.

The e-learning course includes 10 modules explaining each of the best practices. The course includes practical tips and example techniques to use the best practices in physical activity conversations with adults with SCI. Table 8.1 provides a description and rational for the course content of each module.

**Table S8:** Description of course content for each module

| **Module** | **Description** | **Rational** |
| --- | --- | --- |
| 1. Spinal cord injury | The module provides a short introduction on what a spinal cord injury (SCI) is. We also discuss the importance of physical activity for adults with SCI. | Introduction to the topic. |
| 1. Physical activity and exercise | This module provides an introduction on key principles of physical activity. We provide definitions and explanations of terms related to physical activity and exercise, such as frequency, intensity, time and type. We also discuss SCI-specific safety issues related to physical activity. | Introduction to the topic. |
| 1. Theory and evidence of the best practices | This module provides an overview of the key theories and evidence that were used to create the best practices. We also introduce what the best practices for SCI physical activity counselling are and why they were created. The module provides a list of key terms and definitions related to these best practices. | Introduction to the topic. |
| 1. How to have a conversation? | This module focuses on the first three best practices for SCI physical activity counselling. These three best practices focus on how to have a conversation about physical activity with your client with SCI. The best practices are:   1. Build rapport and establish a relationship with your client 2. Use a client-centered approach following the spirit of Motivational Interviewing 3. Tailor the support to your client’s motivation for physical activity and their current needs, values, wishes and preferences. | Explain best practices 1-3 focusing on how to have a conversation. The content in this module provides the foundation for a positive counselling experience. |
| 1. The Ask-Tell-Ask technique | This module focuses on a simple technique called Ask-Tell-Ask. This technique uses the spirit of motivational interviewing. The Ask-Tell-Ask technique will help learners to deliver physical activity counselling in an effective and collaborative way. | Provide example techniques and strategies for using best practices 1-3. |
| 1. What should you always discuss with your client? | This module focuses on the three best practices about the content that counsellors should always discuss in a physical activity conversation with their client. These best practices are:   1. Ask about your client's physical activity experiences 2. Understanding your client’s physical activity capability, opportunity and motivation 3. Understand your client's physical activity barriers and work together with your client to develop possible solutions to overcome these barriers | Explain best practices 4-6 focusing on the content that should always be discussed in a physical activity conversation. The module provides example techniques and example questions to discuss the topics related to best practices 4-6. |
| 1. What should you consider discussing? | This module focuses on best practices on topics that counsellors should consider discussing with their clients during a conversation about physical activity. These best practices are:   1. Provide information on physical activity benefits 2. Share the physical activity guidelines for adults with SCI 3. Goal setting and action planning 4. Sharing physical activity examples | Explain best practices 7-10 focusing on the content that counsellors should consider discussing in a physical activity conversation. The module provides example techniques and example questions to discuss the topics related to best practices 7 – 10. |
| 1. Brief action planning | This module focuses on a counselling technique called Brief Action Planning (BAP). It provides an example of how the best practices can be put into practice.  BAP is an evidence-based technique to support behaviour change. Some of the discussed best practices are incorporated in the BAP technique.  In particular, the BAP technique can help counsellors to work together with their client to set a physical activity goal and create an action plan. | Provide an evidence-based example of how to put the best practices into practice. |
| 1. ProACTIVE SCI example | This module provides examples and resources of the SCI ProACTIVE Toolkit. The SCI ProACTIVE Toolkit provides examples of how you could structure a counselling session. | Provide an evidence-based example of how to put the best practices into practice. |
| 1. Closing and resources | This module discusses things to keep in mind when using the best practices. We provide a list of additional resources for further training and reading. | Provide links to additional resources and training opportunity. |
| BONUS: Example counselling videos | This bonus module provides example videos of counselling conversations following the brief action planning technique. | Provides additional learning opportunities. |
| BONUS: Reflection exercise | This module provides reflection exercises related to the example counselling videos. | Provides additional learning opportunities. |

A list of references and resources supporting the content of each module of the course is available on Open Science Framework.

**Description of the format of the e-learning course**

This is an online, self-guided course available via <https://www.sci-counselling.ubc.ca/>. It takes, on average, 2.5 hours to complete the online, self-guided course. This time is excluding the bonus material and engagement in additional (optional) resources that are provided in this training.

**Description of the didactic methods of the course**

The e-learning course follows a learner-centered approach in which learning objectives are provided at the beginning of the course. We used various strategies to create an engaging, self-guided, learner-centered course. Table 8.2 provides an overview of example strategies including a short description for each Universal Design for Learning (UDL) Guideline.

**Table S9.** Overview of the UDL Guidelines and related examples strategies with descriptions used to apply these guidelines in the e-learning course on SCI physical activity counselling

| **UDL Guidelines** | **Example strategy** | **Description** |
| --- | --- | --- |
| 1. **Provide options for Perception** | Provided information in different formats | A combination of written text, short video presentations (with closed-captions and adjustable speed), figures are used to present the information. Key figures include audio recordings explaining key concepts, other figures include short descriptions. |
| 1. **Provide options for Language & Symbols** | Included introduction modules with key terms and definitions | Modules 1 – 3 include explanations and definitions of key terms, including spinal cord injury, counselling, exercise and physical activity. |
| 1. **Provide options for Comprehension** | Provided the information in a logical order | Modules 1 – 3 are introduction modules with required background information on key topics, modules 4 – 8 explain the best practices and modules 8 – 9 provide practical counselling examples of how to use the best practices, including counselling flow charts. |
| 1. **Provide options for Physical Action** | Used different types of navigation and interaction components | Knowledge checks are incorporated throughout the course. Modules 4 – 7 include summaries of the content presented in flash cards, in which learners can click on the card to read the summary of each best practices. |
| 1. **Provide options for Expression and Communication** | Not applicable |  |
| 1. **Provide options for Executive Functions** | Providing a visual overview of the course outline throughout the course | This visual (refer to Figure 3 in the main text) can guide the learning journey. It provides learners the opportunity to check where they are at in terms of achieving the learning objectives. |
| 1. **Provide options for Recruiting Interest** | Making the course content relevant to learners by engaging end-users throughout the development process and incorporating counselling examples and quotes | The course was developed using input from >160 potential end-users and experts to ensure the course content aligns with needs and preferences of potential end-users. The example counselling videos and examples of counselling questions that learners can relate to make the course relevant and useful to the learners. To ensure the content of the course is relevant and useful to learners, we engaged a diverse group of potential end-users throughout the development process of the course (refer to main text). |
| 1. **Provide options for Sustaining Effort & Persistence** | Providing feedback and explanations after each quiz and knowledge test | The feedback provided after each correct or incorrect answer provides an additional check for learners to check if they understood the content or not and decide whether or not they wish to revisit the course content. |
| 1. **Provide options for Self Regulation** | Including quizzes and knowledge checks within and between modules | The quizzes and question knowledge checks incorporated throughout the course provide learners the opportunity to test their knowledge and understanding of the content. |

*Notes:* UDL Guidelines refer to the Universal Design for Learning Guidelines version 2.2 <http://udlguidelines.cast.org>

**Target audience of the e-learning course**

The course was created for anyone who provides professional guidance or counselling to adults with SCI on starting, changing and/or maintaining a physically active lifestyle. This includes physical activity/lifestyle/health counsellors, SCI peer mentors, occupational therapists, therapeutic recreation professionals, physiotherapists, psychomotor therapists, social workers, kinesiologists, rehabilitation assistants, SCI caregivers, fitness trainers or other coaches.

**Appendix 9:** Results of secondary outcome measures

**Table S10:** Usability, learning experiences, and satisfaction levels

|  | **Mean ± SD (ranges)** |
| --- | --- |
|  | Total group (n=36) |
| **Learning experiences** |  |
| ***User engagement*** | **6.2 ± 0.7 (3.2 – 7.0)** |
| - The online training enabled an active learning environment. | 5.5 ± 1.2 (1.0 – 7.0) |
| - The online training used a variety of learning methods. | 6.0 ± 0.8 (3.0 – 7.0) |
| - The online training had a visually appealing experience. | 6.1 ± 1.1 (2.0 – 7.0) |
| - The online training offered an enjoyable experience. | 6.1 ± 1.0 (2.0 – 7.0) |
| - I enjoyed the overall experience of the online training. | 6.2 ± 0.7 (4.0 – 7.0) |
| - I felt discouraged while doing the online training. | 1.4 ± 0.6 (1.0 – 3.0) |
| ***Technical experience*** | **3.0 ± 2.2 (1.0 – 7.0)** |
| - I experienced technical difficulties while doing the online training | 3.0 ± 2.2 (1.0 – 7.0) |
| **Satisfaction levels** | **6.3 ± 0.7 (4.6 – 7.0)** |
| - I am satisfied with SCI physical activity counselling training modules | 6.3 ± 0.9 (3.0 – 7.0) |
| - I am satisfied with the introductory modules on SCI and physical activity (Modules 1-3). | 6.4 ± 0.8 (4.0 – 7.0) |
| - I am satisfied with the modules on how to have a conversation about physical activity (Modules 4-5). | 6.4 ± 0.6 (5.0 – 7.0) |
| - I am satisfied with the modules on what to say during a conversation about physical activity (Modules 6-7). | 6.3 ± 0.7 (4.0 – 7.0) |
| - I am satisfied with the modules on the counselling examples about brief action planning and the ProACTIVE SCI toolkit (Modules 8-9). | 6.2 ± 0.8 (5.0 – 7.0) |
| - I am satisfied with the additional information and resources provided at the end of the training (Modules 10 and BONUS material). | 6.2 ± 0.8 (4.0 – 7.0) |
| - I am satisfied with the format of the online training modules. | 6.1 ± 1.1 (3.0 – 7.0) |
| - I am satisfied with my overall experiences taking the online training. | 6.4 ± 0.8 (4.0 – 7.0) |

*Notes:* *Learning experiences were reported using two constructs: ‘user engagement’ and ‘technical experience’. User engagement was assessed with 6 items and technical experience was assessed using 1 item. Satisfaction levels were assessed using 8 items. User engagement, technical experience and satisfaction items were assessed on 7-point Likert Scale. Cronbach alfas were: user engagement: 0.8; satisfaction: 0.9.

**Table S11:** Feasibility of the training

| **Feasibility of the training** | **Mean ± SD (ranges)** |
| --- | --- |
|  | Total group (n=36) |
| **Affordability*** |  |
| - Completing the SCI Physical Activity Counselling training is **cost-effective.** | 6.8 ± 0.5 (5.0 – 7.0) |
| - The SCI Physical Activity Counselling training is **affordable** to complete. | 7.0 ± 0.0 (7.0 – 7.0) |
| **Practicability** |  |
| - The online training is **easy** to complete. | 6.4 ± 0.6 (5.0 – 7.0) |
| - The online training requires the **right amount of time** to improve my understanding about best practices for SCI physical activity counselling. | 6.0 ± 1.1 (2.0 – 7.0) |
| **Effectiveness** |  |
| - The online training will be **effective** in improving physical activity counselling for people with spinal cord injury. | 6.2 ± 0.7 (5.0 – 7.0) |
| - The training will be **effective** in improving physical activity levels in adults with spinal cord injury. | 6.0 ± 0.7 (4.0 – 7.0) |
| **Acceptability** |  |
| - The online training would be **acceptable** to be completed by counsellors and other individuals providing physical activity counselling support to people with spinal cord injury | 6.4 ± 0.7 (5.0 – 7.0) |
| - The online training is **suitable** for counsellors and other individuals providing physical activity counselling support to people with spinal cord injury. | 6.2 ± 0.9 (2.0 – 7.0) |
| **Safety*** |  |
| - There are no **side-effects/safety** concerns associated with completing the SCI Physical Activity Counselling training. | 6.9 ± 0.2 (6.0 – 7.0) |
| **Equity*** |  |
| - Individuals from different settings (e.g., healthcare, community, university) would have **equal opportunity** to benefit from the SCI Physical Activity Counselling training. | 6.8 ± 0.4 (5.0 – 7.0) |

*Notes*: All items were rated on a 7-point Likert scale: 1 = strongly disagree, 2 = disagree, 3 = somewhat disagree, 4 = neither disagree nor agree, 5 = somewhat agree, 6 = agree, 7 = strongly agree. *The items on affordability, safety and equity were asked during the interview session. Before asking the items on affordability the interviewer explained to the participants that the training will become freely (no cost) available for everyone after the study has been completed. Cronbach alphas: Practicability: 0.4; Effectiveness: 0.9; Acceptability: 0.8. Cronbach alpha could not be calculated for Affordability due to lack of variation in participants’ responses to the item “The SCI Physical Activity Counselling training is affordable to complete” (i.e., all participants strongly agreed (=7) with this statement).

**Table S12**: Capability, Opportunity and Motivation for using the SCI physical activity counselling best practices

| **Demographics and expertise/background** | **Mean ± SD (ranges)** |
| --- | --- |
|  | Total group (n=36) |
| **Capability** |  |
| - I know how to use the best practices in a conversation about physical activity with my client with SCI following the spirit of Motivational Interviewing. | 5.7 ± 0.7 (3.0 – 7.0) |
| - I know what to discuss during a conversation about physical activity with my client with SCI. | 5.9 ± 0.7 (5.0 – 7.0) |
| - I am confident in my ability to use the best practices in a conversation about physical activity with my client with SCI following the spirit of Motivational Interviewing. | 5.8 ± 0.7 (4.0 – 7.0) |
| **Opportunity** |  |
| - I have the resources (e.g., time and money) to use the best practices in a conversation about physical activity with my client with SCI. | 5.6 ± 1.1 (2.0 – 7.0) |
| - I have support from others to use the best practices in a conversation about physical activity with my clients with SCI. | 5.7 ± 1.0 (2.0 – 7.0) |
| - The decision to use the best practices in a conversation about physical activity is beyond my control. | 2.3 ± 1.4 (1.0 – 6.0) |
| **Motivation** |  |
| - I think there is value in using the best practices in a conversation about physical activity with my client with SCI. | 6.6 ± 0.5 (6.0 – 7.0) |
| - I think it is my responsibility to use the best practices in a conversation about physical activity with my client with SCI. | 6.4 ± 0.6 (5.0 – 7.0) |
| - I intend to use the best practices in a conversation about physical activity with my client with SCI. | 6.4 ± 0.7 (4.0 – 7.0) |

*Notes:* All items were rated on a 7-point Likert scale: 1 = strongly disagree, 2 = disagree, 3 = somewhat disagree, 4 = neither disagree nor agree, 5 = somewhat agree, 6 = agree, 7 = strongly agree.

**Appendix 10**: Summary of findings of interviews with participants in the randomized controlled trial

A total of 36 interviews were conducted with participants who completed the e-learning course. All interviews were conducted over Zoom and took between 15 – 30 minutes. In general, participants were very positive about the course. The felt engaged and said they enjoyed completing the course. All participants would recommend the course to others. Participants also had suggestions to improve the course. Participants shared their perceptions and opinions about the content, the format, length usability, inclusivity and accessibility. They also talked about things they learned from the course and to whom they would recommend it to.

**Content of the course**

Participants mentioned that the content was easy to understand and relevant to them. Some participants mentioned that they already applied some of the things they learned in their daily practices. They also mentioned that most content of the course was also useful for conversations with clients without a SCI. Some participants who were very experienced with working with people with SCI mentioned that it was a good refresher about how to have a conversation about physical activity with their clients.

Participants learned different things. For many participants, the COM-B model was a new thing they learned. They thought it was an easy and useful model to identify their client’s physical activity barriers. Participants also mentioned that they learned to respect their client’s choices and that some clients may not want to talk about physical activity or change their physical activity levels. Participants indicated that in the past they may have pushed these clients more. Participants who had less experienced with working with SCI indicated that after the course they felt more comfortable to talk with individuals with SCI about physical activity. Participants with a research background indicated that this course would be very helpful for everyone who is doing SCI research.

Participants also provided specific suggestions about things that could be improved. In general, suggestions were small details or minor changes. For example, some participants indicated that there were some typos and grammatical mistakes that should be corrected. Some participants indicated that they wanted to have more example counselling videos of a session with clients who are not yet ready to change their physical activity levels. Some participants had specific comments or preferences on terminology that was used in the course.

**Format of the course**

Almost all participants indicated that they liked the format of the course in terms of the combination of short text, short videos and quizzes. They also liked the layout of the course. They said the course looked beautiful. Some participants mentioned that they liked that there were audio-recordings for some of the figures. They indicated that the course provided different ways of presenting information.

Participants also had some comments about format and mode of delivery. Various participants mentioned that there was one speaker in the videos that talked very slow. However, they liked that there was an option to speed up the video. Some participants also indicated that a workbook would be helpful so that they could make notes while going through the course. They said that it would make them feel more engaged. A few participants indicated that they would like to have an option to engage with other learners via a discussion forum, online-sessions and/or practice sessions. Some of them also mentioned that they would like to have an opportunity to practice their counselling skills. However, these participants also acknowledged that there were links to other training opportunities, which they appreciated.

**Length**

Overall participants liked the length of the course. It covered enough information, but it also did not take too much time to complete the training. The estimated 2.5 hours seemed to be reasonable for most participants. Some people took a bit longer to complete the course, others went through it in a shorter amount of time.

**Usability**

Almost all participants indicated that it was very easy to go through the training. They said it was very user-friendly. One participant felt a bit confused when going through the module in which there were YouTube videos. They said they did not know how to turn off the videos and felt a bit lost in that part of the module.

While participants liked that their progress was tracked, this feature was not always properly working (see main text). Some participants felt frustrated or annoyed that they had to re-do the quiz and scroll through the modules to continue with the course.

**Inclusivity and accessibility**

Most participants did not have specific concerns or suggestions about the inclusivity or accessibility of the course. Participants mentioned the plain language that was used throughout the course was accessible for a broad group of learners with various expertise. Some participants indicated that adding closed captions to the videos would improve accessibility of the course.

1. Hoekstra, F., Gainforth, H. L., Broeksteeg, R., Corras, S., Collins, D., Gaudet, S., Giroux, E. E., McCallum, S., Ma, J. K., Rakiecki, D., Rockall, S., van den Berg-Emons, R., van Vilsteren, A., Wilroy, J., & Martin Ginis, K. A. (2023). Theory- and evidence-based best practices for physical activity counseling for adults with spinal cord injury. The journal of spinal cord medicine, 1–13. <https://doi.org/10.1080/10790268.2023.2169062> [↑](#footnote-ref-1)
2. Anderson LW, Krathwohl DR, Bloom BS, Bloom BS (Benjamin S. A Taxonomy for Learning, Teaching, and Assessing : a Revision of Bloom’s Taxonomy of Educational Objectives / Editors, Lorin W. Anderson, David Krathwohl ; Contributors, Peter W. Airasian et al. Complete ed. Longman; 2001.

   Bloom, B. S.; Engelhart, M. D.; Furst, E. J.; Hill, W. H.; Krathwohl, D. R. (1956). Taxonomy of educational objectives: The classification of educational goals. Vol. Handbook I: Cognitive domain. New York: David McKay Company. [↑](#footnote-ref-2)
